# Patient Perceptions of Audio-Only Versus Video Telehealth Visits: A Qualitative Study Among Patients in an Academic Medical Center Setting

**DOI:** 10.1089/tmr.2023.0065

**Published:** 2024-04-03

**Authors:** Ryan Kruis, Elizabeth A. Brown, Jada Johnson, Kit N. Simpson, James McElligott, Jillian Harvey

**Affiliations:** ^1^ Center for Telehealth, Medical University of South Carolina, Charleston, South Carolina, USA.; ^2^ Department of Health Sciences and Research, Medical University of South Carolina, Charleston, South Carolina, USA.; ^3^ School of Community and Environmental Health, Old Dominion University, Norfolk, Virginia, USA.; ^4^ Department of Healthcare Leadership and Management, Medical University of South Carolina, Charleston, South Carolina, USA; ^5^ Department of Pediatrics, Medical University of South Carolina, Charleston, South Carolina, USA.

**Keywords:** telehealth, telemedicine, technology, audio-only, telephone visits

## Abstract

**Introduction:**

Telehealth utilization surged during the COVID-19 pandemic, offering expanded health care access. Audio-only visits emerged as a crucial tool for patients facing technology or connectivity barriers to still use telehealth. This qualitative study aims to better understand patient perceptions of audio-only versus video telehealth visits during the COVID-19 pandemic, and how patients perceive the role of each in their overall health care.

**Methods:**

Semi-structured interviews were conducted with 14 adult patients seeking care at an academic medical center located in the Southeast region of the United States. Patients had experienced both an audio-only and video telehealth visit within the past 6 months. Topics covered in the interview included comfort, preference, quality, and communication during each type of visit. Interviews were transcribed verbatim, coded, and analyzed using a general inductive approach.

**Results:**

Participants valued having both modalities available largely due to convenience and saw these visits as supplemental or supporting their in-person care. Preferences for visit types were varied among participants and were context-specific, influenced by visit purpose and provider rapport. Patients viewed audio-only visits favorably for informational follow-ups and highlighted their convenience, particularly for multitasking and caregiving duties. In contrast, video visits were seen as more effective for communication due to visual cues and better suited for demonstrating health conditions. Audio-only visits were also seen as less technology-dependent and served as a vital back-up to failed video encounters.

**Discussion:**

Despite varied preferences, patients perceived both modalities as complementary to in-person care. Concerns around the quality of care were mitigated by patients' and providers' judicious use of visit types based on clinical appropriateness and existing rapport. The results emphasize the necessity and flexibility of audio-only visits in ensuring equitable access to telehealth, especially for those with technology limitations or demanding responsibilities. To maintain the access and convenience afforded by telehealth and ensure these benefits are offered equitably, policy makers and health care organizations must continue to provide flexible telehealth options, including audio-only visits.

## Introduction

Telehealth, which describes the use of electronic information and telecommunication technologies to support long-distance clinical health care,^1^ has long been used to enhance access to care.^2–4^ Its use skyrocketed during the COVID-19 pandemic, which brought the removal of prior regulatory and reimbursement barriers to telehealth, as policy makers sought to maintain health care access at a time when social distancing was mandated and the health care system was severely strained.^5–7^ While telehealth utilization rates have declined since the initial months of the pandemic, health systems continue to provide a significant number of visits virtually.

According to FAIRHealth, telehealth has continuously accounted for approximately five percent of all claims throughout 2021 and 2022.^8^ Moreover, when surveyed by the American Medical Association, the majority of physicians indicated that their organization was motivated to continue to use telehealth and that patients using telehealth have better access to care.^9^

As part of the telehealth flexibilities, audio-only visits—that is, telehealth visits without a video component often conducted over the telephone—also became permissible and reimbursable during the pandemic. Audio-only visits have proved to be a critical tool for maintaining access to health care for those lacking the adequate technology, internet connectivity, or digital literacy required for a successful telehealth video visit.^5,7^ FAIRHealth estimates that close to 5–6% of all telehealth claims in the first half of 2023 were audio-only.^8^

Among Medicare beneficiaries, the Bipartisan Policy Center estimated that ∼10% of all Medicare beneficiaries had at least one audio-only visit in 2021, with 1 in 5 telehealth services being delivered by telephone.^10,11^ Research on telehealth use during the pandemic has shown disproportionately higher rates of audio-only visits among Black and Hispanic patients as compared with white,^12–15^ patients who are dually eligible, nonprimary English speaking, and rural,^12,14^ patients with low socioeconomic status,^13,16^ and older patients.^14,15,17^

While audio-only visits have helped maintain access, particularly among minoritized or underserved communities already facing barriers to health care access, some providers and policy makers have questioned the quality of such visits in comparison to video visits, noting the importance of visual cues for communication and rapport as well as evaluation when applicable.^11,12,18^ This concern about quality has raised questions regarding health equity. While audio-only visits may extend the benefits of telehealth to lower-resourced and less tech-savvy individuals, if these visits are of lower quality, then their continued promotion may exacerbate health inequities. While some research has shown audio-only to be non-inferior to other visit types for some use cases,^9,19^ continued research on the quality, effectiveness, and experience of audio-only visits is necessary.

While the literature on audio-only visits is growing, most has focused on utilization among different populations and clinic types.^12–14,16,17^ While some research has examined provider perceptions on the value of audio-only visits,^9,20,21^ remarkably absent from the research are the voices of patients. One qualitative study among veterans examining experiences with video visits documented patient value in being able to communicate visual cues over video but also noted feeling “more awkward” and facing significant technological barriers using video.^22^ While other qualitative studies have looked specifically at telephone interventions for certain use-cases,^23–26^ research exploring how patients compare their experience of video versus audio-only visits is remarkably scant.

The aim of this qualitative study was to understand and describe how patients receiving care at an academic medical center in the Southeast perceive the care they received in audio-only versus video telehealth visits as well as the role they see each playing in their overall health care.

## Methods

### Setting

This study took place at the Medical University of South Carolina (MUSC), an academic medical center located in South Carolina, as part of a broader evaluation of MUSC's COVID-response efforts funded by the Agency for Healthcare Research and Quality (R01 HS028284). Like most health systems, MUSC implemented widespread direct-to-patient video visits during the COVID-19 pandemic, which has been documented elsewhere.^27,28^

At the time of this research, MUSC had recently transitioned its clinics to a new telehealth platform with enhanced electronic medical record integration and new features supporting the visit process and workflow. Before this, a simpler, unintegrated video platform was leveraged to accommodate the rapid growth of telehealth during the early days of the pandemic. In addition, MUSC's approach to telehealth visit scheduling and patient support was decentralized and varied greatly across MUSC departments and service lines at the time of these interviews.

### Design

The research team conducted semi-structured, qualitative interviews using a criterion sample^29^ of patients who had had both audio-only and video telehealth visits within the preceding 6 months of January–June 2022. This research was approved by MUSC's Institutional Review Board and performed and presented in accordance with the Consolidated Criteria for Reporting Qualitative Research checklist.^30^

### Recruitment

A total of 290 patients were identified in the electronic medical record as having had at least one audio-only and video visit in the past 6 months. Of these, a random sampling of 50 white patients and 50 non-white patients were identified for cold contact recruitment to participate in the study. Non-white patients were oversampled in efforts to ensure representation given their documented higher rate of audio-only visit utilization compared with white counterparts.

All 100 patients were cold contacted using both phone numbers and email addresses listed in their medical records. Participants were offered a $20 gift card to participate in the interview. Interviews were conducted until saturation was reached, which was determined through study team consensus that interviews produced redundant information.^29^ A total of 14 individuals agreed to be interviewed from the cold contract recruitment methods.

### Data collection

All interviews were conducted by authors R.K., E.A.B., and J.J., all of whom are health services researchers with training in qualitative methods. A semi-structured interview guide was developed to facilitate interviews (Supplementary Data). Topics included: general health care use, reason for and referral process for telehealth visits, experiences with both video visits and audio-only visits, comparison of audio-only and telehealth visits across numerous topics (i.e., comfort, engagement, professionalism, privacy, ability to get needs met), and long-term preferences for telehealth visit types.

Verbal consent by participants was provided before beginning the interviews. All 14 interviews were conducted during 2 months in Fall 2022, with the average length of interviews being 18.75 min (range: 9.5–27.0 min). All interviews were conducted over the phone (i.e., audio-only), were professionally recorded, and were transcribed verbatim. Additional recruitment was not necessary as data saturation was reached after the 14 interviews.

### Data analysis

All raw textual data from the transcribed interviews were analyzed using a general inductive approach.^31^ A general inductive approach is guided by the evaluation objectives, but findings arise directly from analysis of raw data not *a priori* expectations or models. Two authors (R.K. and J.J.) each independently coded two interviews using open coding methods and then met with the rest of the study team (E.A.B. and J.H.) to discuss common codes and develop an initial codebook. The initial codebook was independently applied to two additional interviews to determine whether further refinement was needed (R.K. and J.J.).

After this step, the codebook was finalized (Table 1) and applied to all 14 interviews. Each transcript was coded twice using the final codebook (R.K., J.J., E.A.B., and J.H.); any discrepancies in codes were discussed by the study team to reach consensus. All coding took place using NVivo 14 (Luminero. Denver, CO, USA) qualitative coding software.^32^

**Table 1.** Code Book

Category	Code name	Definition
**Visit Codes:** All statements related specifically to a type of visit should include a visit type code (e.g., audio-only visit, video visit, telehealth visit, in-person visit)	Audio-only visit	Statements referring to an audio-only visit specifically
	Video visit	Statements referring to a video visit (audio + video) specifically
	Telehealth visit	Statements inclusively referring to both video visits and audio-only visits together
	In-person visit	Statements referring to visits that occur in person
**Attitude Valence Codes:** To be used with visit type to indicate the preference/dislike of that visit type. (e.g., audio-only +). For example, a statement regarding liking video visits because of ease of communication would be: “video visit +, communication”	+ (positive)	Any statement about a visit type with a positive valence or indicating a positive preference
	− (negative)	Any statement about a visit type with a negative valence or indicating a dislike
**Experience Domains:** Topics that arose from open coding of interviews related to visit type and experience. Some of these domains were specifically asked about in the interview guide	Convenience	Any statement about a visit being more or less convenient or time saving due to travel time, wait time, routine interruptions, etc.
	Comfort	Any statement about a visit being more or less comfortable for a patient to participate in. Refers to the emotional state of the patient
	Technology	Any statement regarding technology of either type of telehealth visit. Failed Visit: Subcode of technology; used when patient describes a visit failing due to technology
	Provider dynamics	Any statement regarding perceptions of the provider during a visit and their behavior (e.g., provider being more or less rushed, more or less interested, etc.). This could also refer to relationship with provider influencing decisions around visits
	Privacy	Any statement regarding privacy aspects of visits
	Communication	Any statement regarding the communication abilities and/or comprehension during a visit; includes mention of visual cues to aid comprehension
	Visit purpose	Any statement regarding preference based on the content or purpose of the visit (e.g., medication management, test results, hands-on evaluation)
	Infection control	Any statement regarding COVID mitigation or other mitigation of sickness as a benefit (or not) of telehealth
	Scheduling visit	Any statement about how a telehealth visit was scheduled or initiated
	Medical condition	Any statements regarding a patient's medical condition (e.g., chronic disease, comorbidities, acute condition like cancer, etc.)
	Provider type	Any statements regarding the type of specialist or provider

R.K. wrote summaries for all codes and selected illustrative quotes for each summary; these summaries were reviewed and edited by the study team (J.J., E.A.B., and J.H.). Finally, consistent with the general inductive approach, the study team reviewed all summaries to: (a) identify cross-cutting themes most pertinent to the study's evaluation objectives and (b) develop a table of participant identified “pros” and “cons” to audio-only versus video telehealth visits.

## Results

### Participants

The demographics of the 14 patients who participated in the study are outlined in Table 2. The mean age was 52.9 years (±10.1 standard deviation), with the youngest participant being 29 and the oldest 64. Participants were predominantly female (71%) and white (86%), despite efforts to over sample non-white patients. Notably, 11 participants (79%) lived outside of Charleston County, which is where the bulk of MUSC's specialty or tertiary care providers are located. In terms of payor coverage, half of the participants were commercially insured, 3 (29%) had Medicare coverage, and 4 (29%) had Medicaid coverage. Participants noted seeing multiple providers via telehealth: the majority indicated using telehealth to see one or more medical specialists for chronic illnesses (71%), whereas fewer numbers indicated using telehealth for their primary care (21%) or behavioral health (21%).

**Table 2.** Participant Demographic Characteristics

Characteristic	Participants (***n*** = 14), ***n*** (%)
Age, years, mean ± SD	52.9 ± 10.1
Gender	
Male	4 (29)
Female	10 (71)
Race	
White	12 (86)
Black or African American	2 (14)
Geography	
Charleston County Residents	3 (21)
Primary insurer	
Commercial	7 (50)
Medicare	3 (21)
Medicaid	4 (29)
Identified telehealth providers	
Specialty care	10 (71)
Primary care	3 (21)
Behavioral health	3 (21)

SD, standard deviation.

### Patients want choices for telehealth visits

One of the most prominent themes across interviews was that patients value having choices for telehealth, including having both video and audio-only modalities available. According to one respondent, “We had never done [telehealth] before 2020 and wouldn't even have imagined doing it. And I think we've both, kind of given the right circumstances, would jump at a chance to take the telehealth visit over the in-person visit” (P 112). Patients were divided and nuanced in terms of their preferences for video versus audio-only telehealth visits, with decisions for each being largely situational and based on the visit purpose, rapport with provider, and convenience.

#### Visit purpose

Many participants noted the reason for the visit as the driver determining their preference for and the appropriateness of telehealth. Telehealth visits, and particularly audio-only telehealth visits, were seen as ideal for required follow-up visits that were largely informational in nature (e.g., prescription refills, receiving results from labs or imaging).

One patient noted, “She [the provider] offered me the telehealth and I jumped at it because it wasn't necessary for me to be physically examined; it was just necessary to check in and make sure things were as expected…It's completely unnecessary for it to be video” (P 112). Participants who generally preferred telehealth acknowledged that some conditions required “hands-on” assessment or in-office lab work thus necessitating in-person visits. “I don't normally have video visit with my rheumatologist. I go see him. I have to literally go see him, because I have to have blood work done, and all that other stuff” (P 110).

When comparing video versus audio-only telehealth visits, multiple participants noted the importance of video when needing to demonstrate something visually to a provider. “The video visits are important, obviously, if I had something to show whether it was what part of my head was hurting or a rash that I had. Sometimes I would show them some exercises that I was doing to make sure that they were okay with the exercises” (P 106).

#### Provider rapport

Rapport with providers was another large factor influencing participants' situational visit preferences. Numerous participants indicated feeling no differently about their engagement with their provider in telehealth versus in-person, explaining this in the context of having known their provider for multiple years.

I think audio is the best thing….Like I said, especially with a doctor that I know, that I've been seeing and so forth and so on, I don't see any reason why I need to see her in a video, you know? I don't see any reason to. You know, if it were a new provider or—then I would probably say I want a video visit. (P 104)

Others indicated a stronger preference for in-person or video visits, as they felt visually seeing their provider facilitated better rapport building. Participants indicated this as especially important when working with new providers. For example, two different patients stated,

I just felt that they would take me more seriously if they realized I was a real person. If they could see me, see me struggling, see me focused on solving this, I was hoping that they would take me more seriously. And if I needed them in the future, that they would be more responsive… I do think seeing the person helps establish more of a relationship than just hearing them. It turns it into a two-dimensional relationship instead of just a one-dimensional audio. (P 106)

[A video visit] is like being in the office with [my therapist]. Me and her, she's been my counselor for, ooh, some years now…So when I get on video with her, it's like I just get so excited. She's happy to see my face, and I'm happy to see hers. And it's just a big difference. It's a difference to me than you just being on the phone, me just hearing your voice. You gotta have a face to it. (P 110)

Notably, two participants noted perceiving their providers as less “rushed” during telehealth visits as compared with in-person visits. “Well, with the video visit I actually…I feel like there's more time, that I've got more time because she has time set that she's outside of her—not in her office in the days that she does the video visits. So, I actually feel that there is more time available” (P 114).

#### Convenience

Nearly all participants noted that telehealth visits were generally more convenient than in-person visits, and thus was one of the most prominent factors influencing patient preference. “I don't really think there was anything I didn't like. I like being able to not have to leave my house, not have to park, not have to, you know, worry about the COVID, the germs, all that. It was really convenient for me” (P 107). Reasons included telehealth being more efficient in terms of time (i.e., no drive times, difficulty navigating city traffic or parking, not having to wait in the office) and participants not having to disrupt daily responsibilities (e.g., work, childcare, walking dog).

You don't have to drive to get there. You don't have to make any other arrangements while you're gone, take care of maybe—we have a dog. So, pretty much you just stay right there at the house and do your visit… it's—like I said, you don't have to leave the house if you don't want to and drive in traffic, and then try to find a park, and then go to your visit, then get back, fight traffic, and get back home. And that makes it nice. (P 101)

### Comparing video and audio-only telehealth visits

While participants were varied in terms of their overall preferences for video versus audio-only telehealth visits, common themes arose regarding the pros and cons of each modality across the domains of technology, comfort, communication, convenience, and privacy.

#### Technology

Multiple participants reported ease or no issues with using the telehealth technology. “I think the software was pretty self-explanatory, as far as the visit goes. Logging on was easy” (P 101). Others, however, spoke at length about technology challenges.

Sometimes it won't connect at all. You won't get no voice. You won't get no picture. Or you'll get a picture, no voice. I don't know. And then like I said, I've been introduced to three different telehealth things. I don't know which one to connect to. You know? When to connect to this one with this doctor. So I don't know. It's just, it's very, very helpful. It's convenient, now. Don't get me wrong. It's just that it's so inconvenient when the Internet doesn't connect. (P 110)

Challenges included: navigating multiple links, different platforms being used across providers and over time, and connection issues. Some participants indicated that technology issues they experienced had “smoothed out” over time as providers and patients became more acquainted with telehealth.

I would say half the time everything worked on both sides. Between my video, the doctor's video, my audio, the doctor's audio, my mic, the doctor's mic. I think in the beginning, things were a little bit awkward to try to get everything configured correctly. And then it just seemed that as time went on, everybody just got more familiar with it… it all just kind of smoothed itself out throughout last year. (P 106)

Notably, more than half of the participants (*n* = 8) indicated that their audio-only visits were a result of a failed telehealth video visits, during which either the provider or patient suggested moving the visit to audio only.

#### Comfort

More than half of the participants voiced no difference in terms of their comfort levels when comparing audio-only with video visits. “I mean I feel just as comfortable with a video or on a call than I do in office” (P 113). Two individuals noted feeling more comfortable with audio only visits because they did not have the stress related to the technology working, did not have to worry about their appearance, and felt “less on the spot” (P 103). According to one patient,

The one thing that was actually nice about the phone visit, phone only, was the doctor would call me, so I didn't have to hunt around to find the right link to click on. And once I confirmed that he was going to call my cell phone, I could relax and say okay, I don't schedule any appointments during that hour and I could sit here and wait for the phone to ring. And when they called my cell phone, it was perfect and the least stressful of all because I didn't have to do anything… The telephone is easier, less stressful because you don't have to worry about technology problems. (P 106)

For those feeling more comfortable with video visits, this was typically related to the rapport they felt with the provider or strengthened ability to communicate due to being able to see their provider visually. “I liked the video visits, especially for sight. I like the video visits because, I don't know, it's just like being in the office with them….To me it just made me feel more secure” (P 110).

#### Communication

Participants overwhelmingly considered communication more effective over video visits as compared with audio-only visits. Participants discussed two main reasons for this. First, participants noted that video visits allow for communication via facial queues and other nonverbal means that enhance the experience.

I do think it's slightly more favorable to be able to see the person's face, and then you can see their expressions or they can see yours. So I think there's some things to see there perhaps there's some benefit to that. You know, is it absolute? You know, can you quantify that? I don't know. But certainly I think this adds a little bit. It's easier…when you see someone's body language and all of that, because I have a better connection and understanding. (P 112)

Second, many participants noted that video visits also allow providers to observe health conditions when needed (e.g., rash, rehab exercise, etc.).

I like the audio/video. But I like the video because I have lupus on my face and my doctor likes to look at it to see—if I can't be there…I like it so he can look at my face and tell how I'm looking…Like my lips—I'm just saying my lips are affected, so it's—and the bottom of my—below my lips. So, I like for him to be able to look at it. (P 108)

#### Convenience of audio-only

As noted previously, convenience was a large factor influencing preference for telehealth in general over in-person visits. When comparing audio-only with video telehealth visits, a number of participants noted audio-only visits being more convenient than video visits due to being able to multi-task (e.g., drive, eat lunch, do laundry), not having to worry about appearance, and being able to maintain daily responsibilities more easily by not requiring video. “[Audio-only visits] are just as convenient. I mean, especially because some of my visits are in the car. I probably shouldn't be doing that. But you know, I'm, I'm a very busy person and you know, so I can chit chat and you don't have to see me” (P 104). Two participants in particular brought up audio-only being more convenient in the context of being a caregiver.

I'd say the audio because like I said, the video, you have to login and then if you're like me, I have kids who—at that time, that would be in the summertime—so I had kids who were home and I have to worry about them, don't come around me while I'm waiting on this video. That's the only thing. The audio was better as far as convenient for me. (P 109)

#### Privacy

Although the interview guide directly asked questions about privacy, notably all patients indicated they had no concerns about their privacy as part of their telehealth visits and felt their privacy was respected in both video and audio-only visits.

### Identified pros and cons of audio-only versus video telehealth visits

Across the thematic domains described earlier, some common positive and negative aspects of both audio-only and video telehealth visits emerged from participant interviews. These are summarized in Table 3.

**Table 3.** Themes from Comparing Video to Audio-Only Telehealth Visits

	Audio-only visit	Video visit
Pros	• Less stressful waiting for an audio-only call (a telephone call) than navigating a telehealth platform. • More convenient for patients. • Useful when getting results back on labs or a procedures. • Fewer technical problems (even those who preferred video, noted appreciated that this was available as a back-up)	• Greater connection between patient and provider. • Non-verbal communication is possible in video visits. • Ability to demonstrate something to the provider
Cons	• Less relational and less able to perceive non-verbal communication. • Limited in what can be addressed	• Technology issues. • Variations in how visits are scheduled and platforms accessed

## Discussion

The proliferation of telehealth since the COVID-19 pandemic has not only increased access to health care but also added significant convenience to the patient experience. Findings from this qualitative exploration of patient perceptions of video versus audio-only telehealth underscore the value consumers place on having options for care, including both video and audio-only options. While divided and varied in terms of preference for telehealth visit types, patients agreed that both video- and audio-only telehealth visits have a place alongside their in-person care.

The study showed that the decisions patients and providers make regarding the use of different telehealth modalities are situational: each has its own pros and cons and may be more or less appropriate given a patient's demographics (e.g., being geographically distant) and the purpose of a visit.

While patient satisfaction with telehealth is well documented,^33–35^ differentiating patient experience as it relates to video visits versus audio-only visits is less common. While some survey research has shown satisfaction can differ slightly between video versus-audio only visits, with patients slightly rating video visits more favorably, these differences are slight.^36,37^ This qualitative research provides important nuance, elucidating themes that may influence patients experience of the care they receive.

This study's findings are also timely as continued payment for audio-only visits is uncertain in the United States. The Centers for Medicare and Medicaid Services have agreed to continue coverage through 2024, after which, barring any policy changes, limitations on audio-only visits will go into effect.^38^ Limitations include limiting audio-only to behavioral telehealth and requiring periodic in-person visits. Other insurers are making similar moves.^39^ As a result, many organizations are calling for continued coverage of telehealth broadly, and audio-only visits specifically, to ensure equitable access to telehealth.^40–42^

These study findings highlight a couple of key considerations for the ongoing discussion around future policy and payment related to telehealth, and specifically audio-only visits. First, when considering telehealth visits generally, many cite concerns about the quality of care provided via these modalities. This study, however, suggests that patients and providers alike are quite thoughtful in terms of determining the clinical appropriateness for a visit type based on the purpose of the visit and pre-existing rapport with a provider.

Participants saw telehealth visits—both video and audio-only—as complements to their in-person care, not replacements. While participants acknowledged not being able to see visual cues as a drawback of audio-only visits, they also acknowledge lack of visual cues may be less important when you have an established relationship with provider or are just receiving lab results. Implementing policies that enable flexibility of visit types might better allow providers and patients to collaboratively craft more patient-centered approaches to health care visits and communication.

Second, the study emphasizes the importance of maintaining audio-only visits to ensure equitable access to telehealth. Over half of the participants indicated having to use audio-only visits as a back-up to failed technology in a video visit. Patients with poor broadband connectivity, inadequate technology, or lower digital literacy are more apt to experience failure with video visits or to not have access to video visits at all.^12,14^

Others mentioned audio-only visits being more conducive to balancing alongside childcare or work duties, suggesting added access to individuals that may not otherwise have time or dedicated space for telehealth. As such, maintaining coverage of audio-only visits both as a back-up option to video and as a primary means of telehealth when appropriate is critical if telehealth access is to be equitable.

Finally, one of the predominant themes across interviews was the appreciation for the convenience provided by telehealth, regardless of modality. Consumer demand for telehealth is likely to continue regardless of whether the payer landscape keeps up. As more companies enter the health care market as “disrupters,” the onus will be upon traditional health care organizations to innovate and adapt to remain competitive and work creatively to offer these visits to consumers. Providing these telehealth visits as part of shared-risk and value-based care arrangements may be one avenue for this innovation.^43–46^

### Limitations

This study has its limitations. First, the study took place among patients seeking care at an academic medical center in the Southeast. As such, findings may be less generalizable to other care settings with fewer specialty and sub-specialty providers or other regions of the country with higher concentrations of health care providers. Moreover, while efforts were made to obtain a racially diverse cohort through over sampling, only two of the 14 participants were unidentified as non-white in the electronic medical record.

While the sample was more diverse in terms of payer coverage and geographic location, the limited racial diversity limits the representativeness of the sample. The study also lacked linguistic diversity; incorporation of interpreters into virtual workflows may hold unique challenges for patient experience, further limiting the generalizability of these findings.

In addition, a large number of patients declined to participate, which may have impacted the findings; the study team, however, did reach saturation of themes, and no new insights or differences by respondent type were noted. Further, participants were intentionally sampled based on having had both video and audio-only visits in the past 6 months to gain perspectives on each. As a result, themes may not represent the perspectives of patients using only video or only audio-only visits. This approach to sampling likely also resulted in oversampling of patients with technology issues, as many audio-only visits result from failed video visits. However, to compare the value of both visit types, it was important to obtain the perspectives of those who had lived experience with both telehealth visit types.

Finally, while the focus of the study was exploring patient experience of different telehealth modalities, clinic approach to visit scheduling and workflows may have just as strong an influence on experience as the modality itself. For example, a provider seeming more or less rushed may have more to do with the clinic's scheduling process than whether the visit was in-person or a certain modality. Future research should examine best practices for integrating telehealth into clinic workflows and any associated impact on patient experience.

Despite these limitations, the themes uncovered in this study comparing patient perspectives on audio-only versus video telehealth visits have great import for the ongoing discussions of the role these visits have in the future of health care delivery.

## Conclusion

In conclusion, patients value having options for both video and audio-only telehealth alongside their in-person care. Decisions around the use and appropriateness of each are situational, often decided on collaboratively among the patient and provider. While video visits were seen as better for communication and able to address more conditions, audio-only visits were more convenient, had fewer issues with technology, and were less stressful to complete.

Moving forward, it is important that practice, policy, and payment align and innovate to support continued access to telehealth services—both audio-only and video—in the most clinically appropriate, cost-effective, and equitable ways.

## Authorship Contribution Statement

R.K.: Conceptualization, methodology, investigation, formal analysis, writing–original draft, and project administration. E.A.B.: Conceptualization, methodology, investigation, formal analysis, and writing–review and editing. J.J.: Investigation, formal analysis, and writing–review and editing. K.N.S.: Supervision, funding acquisition, and writing–review and editing. J.M.: Writing–review and editing. J.H.: Conceptualization, methodology, writing–review and editing, funding acquisition, and supervision.

## Supplementary Material

Supplemental data

## Abbreviations Used

MUSC: Medical University of South Carolina
SD: standard deviation
