# Supplementary material for: Patient Perceptions of Audio-Only Versus Video Telehealth Visits: A Qualitative Study Among Patients in an Academic Medical Center Setting
Source: Telemed Rep. 2024 Apr 3;5(1):89–98. doi: 10.1089/tmr.2023.0065 (PMC11002560; doi:10.1089/tmr.2023.0065)
Supplement: Supplemental data [file Suppl_Data.docx]

**Protocol: Patient Perspectives on Audio-Only Versus Video Telehealth Visits**

**INTERVIEW GUIDE**

Thank you for taking time to do this interview. My name is ___, and I am a researcher at MUSC. We want to learn more about what you think about audio-only telehealth visits—in other words telehealth visits without any video, like over the phone—compared to telehealth visits with video. We reached out to you because you have had experiences with both audio-only and video visits here at MUSC.

Before we get started, we want to let you know that everything we discuss today will be confidential and your name will not be associated with any information you provide. If there are any questions you do not wish to answer, please feel free to let us know and we will move forward. We’ll be taking notes but we’d also like to record our discussion so we can go back to it later.

You should have received an email with information about this study when this call was scheduled. Correct? *(If not, re-send the email, or if not in front of a computer, let the respondent know that you will send the email immediately after the interview).*

Do you consent to participate in this interview?

May I record the interview? *(If someone replies that they do not want to be recorded, stop the recorder and take written notes during the interview.)*

Do you have any questions before we get started?

Focus: General Use of Health Care

1. To start, could you please tell us about your use of health care?
   1. Probe: How often do you have visits with a healthcare provider? This can include virtual or in-person at the provider’s office.
   2. Probe: What kinds of providers are these typically?
   3. Probe: What are the main reasons why you schedule visits with your provider(s)?

Focus: Video Visit Experience

1. Your electronic medical record showed that you had at least one video visit in the past 6 months. Could you tell me about that video visit and your experience? Just a reminder, this is about visits when you saw your provider on the screen.
   1. Probe: Reason for visit
   2. Probe: Who suggested to use a telehealth video visit? (something to get at decision making)
   3. Probe: How was the video visit scheduled?
2. What did you like about the video visit?
3. What did you not like about the video visit?
4. Were there any challenges with the visit? If so, explain.

Focus: Audio Only Visit Experience

1. Your electronic medical record also showed that you had at least one audio-only (phone) medical visit in the past 6 months. Could you tell me about your audio-only visit(s) and your experience(s)? Just a reminder, this is about instances when you did not see your provider on the screen and maybe did your visit over the telephone.

🡪 Note: Redirect to discussion about visits with a billing or scheduling employee if a patient discusses other phone encounters.

- 1. Probe: Reason for visit
  2. Probe: Who suggested to use a telehealth audio-only visit? (something to get at decision making)
  3. Probe: How was the audio-only visit set up?

1. What did you like about the audio visit?
2. What did you not like about the audio visit?
3. Were there any challenges? If so, explain.

Focus: Comparison of Visits

1. How would you compare your experience doing an audio-only versus a video visit.
   1. Probe: Engagement with provider
   2. Probe: Professionalism of provider
   3. Probe: Comfort in the discussion
   4. Probe: Comfort with provider seeing home environment
   5. Probe: Ability for your provider to understand your health condition
   6. Probe: Ability to get your health needs met
2. How would you compare the quality of care you received between audio only and video visits?
   1. Probe: How valuable do you find each type of visit?
3. Do you feel your privacy was respected more with one type of visit? Why/why not?
4. Would you choose one format over the other? Why or why not?
5. What is the best mix for you (sweet spot) for using phone or video telehealth visits for your care needs?
   1. Probe: How would your opinion change if more frequent, shorter visits were an option?
      1. Probe: How would more frequent, shorter visits influence your relationship with your provider, if at all?
6. Is there anything else you’d like to discuss?

Wrap up. Thank you again for meeting with us today.
